# HSF1-Controlled and Age-Associated Chaperone Capacity in Neurons and Muscle Cells of *C. elegans*

**DOI:** 10.1371/journal.pone.0008568

**Published:** 2010-01-05

**Authors:** Andreas Kern, Bianca Ackermann, Albrecht M. Clement, Heike Duerk, Christian Behl

**Affiliations:** Institute for Pathobiochemistry, University Medical Center of the Johannes Gutenberg-University Mainz, Mainz, Germany; University of Arkansas for Medical Sciences, United States of America

## Abstract

Protein stability under changing conditions is of vital importance for the cell and under the control of a fine-tuned network of molecular chaperones. Aging and age-related neurodegenerative diseases are directly associated with enhanced protein instability. Employing *C. elegans* expressing GFP-tagged luciferase as a reporter for evaluation of protein stability we show that the chaperoning strategy of body wall muscle cells and neurons is significantly different and that both are differently affected by aging. Muscle cells of young worms are largely resistant to heat stress, which is directly mediated by the stress response controlled through Heat Shock Transcription Factor 1. During recovery following heat stress the ability to refold misfolded proteins is missing. Young neurons are highly susceptible to chronic heat stress, but show a high potency to refold or disaggregate proteins during subsequent recovery. The particular proteome instability in neurons results from a delayed induction of the heat shock response. In aged neurons protein stability is increased during heat stress, whereas muscle cells show enhanced protein instability due to a deteriorated heat shock response. An efficient refolding activity is absent in both aged tissues. These results provide molecular insights into the differential protein stabilization capacity in different tissues and during aging.

## Introduction

Native cellular proteins possess a generally low stability and are always at risk for denaturation and aggregation. Interestingly, gene expression levels and aggregation rates of the accordant proteins are inversely correlated, indicating that proteins have evolved to resist aggregation and to function efficiently. On the other hand they have almost no margin of safety to respond to changing genetic or environmental factors that challenge their structural integrity *in vivo* [1]. Thus, molecular crowding and changing environmental factors facilitate protein denaturation [2], which results in aggregation and depletion of functional cellular proteins. Protein conformation and stability are controlled by the action of molecular chaperones, which are organized in a complex network and are either constitutively expressed or induced under stress conditions through the activity of the Heat Shock Transcription Factor 1 (HSF1) [3]. Chaperones are typically classified as heat shock proteins (HSPs) and annotated by their molecular weight: HSP100, HSP90, HSP70, HSP60 and small heat shock proteins (sHSPs) [4]. They support folding of polypeptides from synthesis, prevent protein denaturation or aggregation and direct proteins to degradation when refolding fails [5]. It is known that the expression of chaperones varies within different cell-types and during aging [6], [7], but their total capacity and regulation in different tissues is not well-established. Also, the functional capability of the chaperone network in an aged cellular environment *in vivo* is largely unknown.

Aging is associated with enhanced protein aggregation and generation of protein inclusions in virtually all cell types. Interestingly, several age-related neurodegenerative diseases like amyotrophic lateral sclerosis or Parkinson disease are directly associated with protein aggregation in distinct regions of the central nervous system despite the ubiquitous expression of affected proteins. Modification of the chaperon network can be beneficial for disease progression [8]–[10].

To analyze the folding capacity of the chaperone network in different tissues *in vivo*, we established transgenic *C. elegans* that express luciferase C-terminally tagged to GFP (Luc::GFP) in neurons or in body wall muscle cells. Employing these nematodes we analyzed tissue-specific differences in chaperoning, the induction of the HSF1-controlled heat shock response as well as age-associated alterations in the chaperone capacity of neurons and muscle cells.

## Results

### Tissue-specific analysis of Luc::GFP denaturation/aggregation

**Figure 1:**
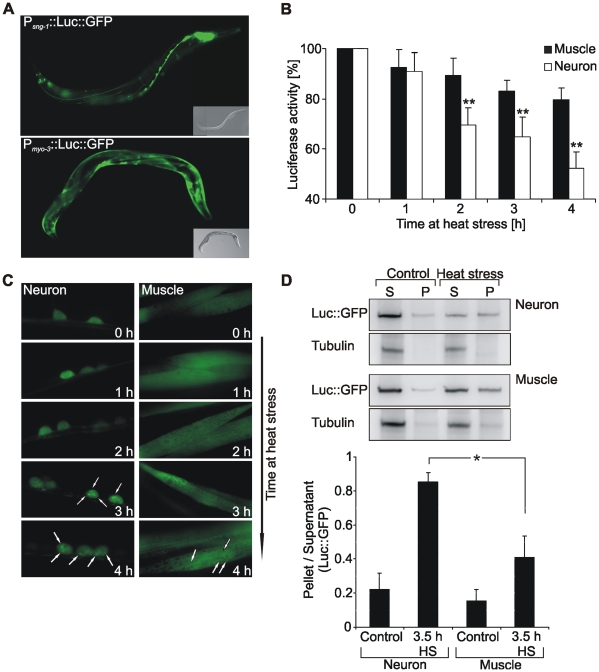
Tissue-specific analysis of Luc::GFP denaturation/aggregation. (A) Fluorescence micrographs of nematodes expressing Luc::GFP in neurons or muscle cells. (B) Analysis of luciferase activity during heat stress at 35°C. Luciferase activity from total lysates of 1 day adult Luc::GFP expressing worms was determined at indicated times of heat stress and compared to unstressed worms. Asterisks represent the statistical significance between muscular and neuronal luciferase activity at a given time point. **P<0.01, Student's *t*-test, n = 5. (C) Fluorescence micrographs of Luc::GFP in neuronal and muscle cells at indicated times of heat stress. Arrows indicate aggregates. (D) Immunoblotting of the soluble (S) and aggregated (P) fraction of Luc::GFP after 3.5 h heat stress. Luc::GFP was detected by an antibody directed against luciferase. Tubulin served for control of the preparation. Graphical representations of the ratio P/S were calculated using optical band densities. *P<0.05, Student's *t*-test, n = 3.

We generated transgenic *C. elegans* expressing Luc::GFP regulated by a promoter for neuronal or body wall muscle cell expression (Figure 1A) and found a tissue-specific stability of the reporter protein upon chronic heat stress. Luciferase activity in neurons was rapidly decreasing during stress, while in muscle cells luciferase activity was rather stable (Figure 1B). This effect was independent of alterations in reporter protein levels, since no heat-induced changes in Luc::GFP protein levels were detected in both tissues (Figure S1). Evaluation of Luc::GFP during heat stress using GFP fluorescence and immunoblotting of aggregates demonstrated that the decreasing luciferase activity in neurons was associated with an increased incidence of aggregates (Figures 1C, D). Aggregate formation was higher in neurons than in muscle cells. Remarkably, the basic cellular concentration of reporter protein was higher in muscle cells (Figure S1), which confirms that the observed instability of neuronal Luc::GFP was definitively not due to tissue-specific differences in expression density of the reporter protein.

The increased sensitivity for denaturation and aggregation of neuronal compared to muscular Luc::GFP suggests that the chaperone network in neurons has a lower capacity for protein stabilization during heat stress.

### Tissue-specific analysis of Luc::GFP recovery/refolding

**Figure 2:**
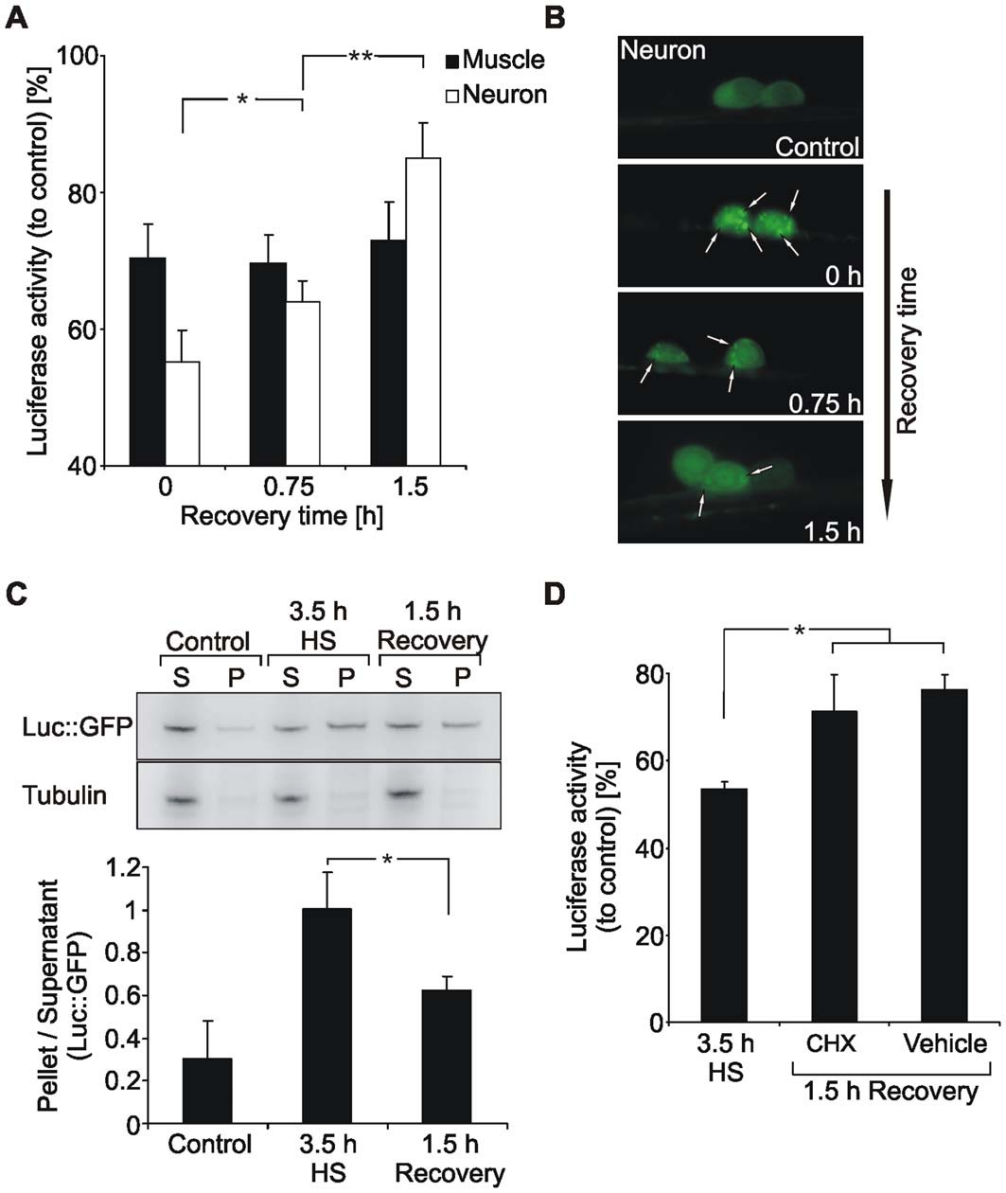
Tissue-specific analysis of Luc::GFP recovery. (A) Analysis of luciferase activity during recovery at 20°C after 3.5 h heat stress. Luciferase activity from total lysates of 1 day adult Luc::GFP expressing worms was determined at indicated times and was compared to unstressed controls. *P<0.05, **P<0.01, Student's *t*-test, n = 5. (B) Fluorescence micrographs of Luc::GFP in neurons at indicated times of recovery at 20°C after 3.5 h heat stress. Arrows indicate aggregates. (C) Immunoblotting of the soluble (S) and aggregated (P) fraction of neuronal Luc::GFP at 3.5 h heat stress and 1.5 h recovery. Luc::GFP was detected by an antibody directed against luciferase. Tubulin served for control of the preparation. Graphical representations of the ratio P/S were calculated using optical band densities. *P<0.05, Student's *t*-test, n = 3. (D) Refolding of Luc::GFP after inhibition of protein translation. Neuronal Luc::GFP expressing worms were heat stressed for 3.5 h at 35°C. During the following recovery period worms were incubated with 0.6 mg/ml cycloheximide (CHX) or vehicle, respectively. Luciferase activity was determined at 3.5 h heat stress and 1.5 h recovery and was compared to unstressed controls. *P<0.05, Student's *t*-test, n = 4.

Denatured or aggregated proteins can be refolded by the action of defined chaperones, such as HSP70, HSP110 and sHSPs [11]. The refolding potential of neuronal and muscle cells was analyzed during a recovery period at 20°C following heat stress (Figure 2A). Interestingly, we found a potent refolding activity in neurons, while a recovery in muscle cells was missing. The enzymatic activity of neuronal luciferase recovered within 1.5 h after heat stress almost back to the level of unstressed worms. The high refolding or disaggregation activity of neurons was supported by visualization of Luc::GFP inclusions and analysis of aggregates by immunoblotting (Figures 2B, C). The recovery was mediated by refolding and not by generation of new reporter protein, since cycloheximide, an inhibitor of protein synthesis, did not affect recovery efficiency (Figure 2D and Figure S2).

Thus, while neurons are rather sensitive for protein denaturation during heat stress, they exhibit a high potential for refolding and disaggregation of misfolded proteins. In contrast, proteins were largely stabilized during heat stress in muscle cells, but a recovery of denatured proteins was missing.

### The increased susceptibility of neurons against heat stress is due to a delayed heat shock response

**Figure 3:**
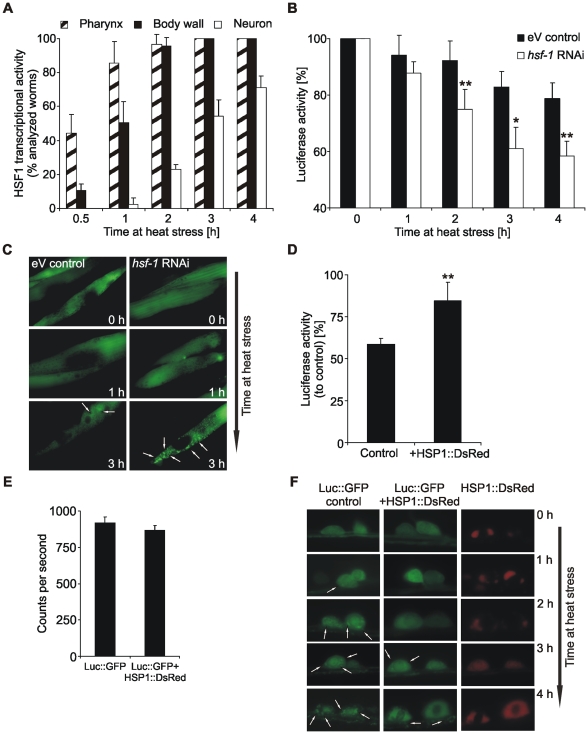
Neurons are susceptible to heat stress due to a delayed heat shock response. (A) Analysis of HSF1 activity, using the expression of P*_hsp-16.2_*::GFP. 1 day adult CL2070 worms were heat stressed for indicated times and the appearance of a GFP fluorescence in different tissues was analyzed in a total of at least 80 worms (n = 4). The temperature for heat stress was reduced to 32°C due to an increased susceptibility of the strain. (B) Analysis of luciferase activity during heat stress at 35°C after *hsf-1* RNAi. Luciferase activity from total lysates of muscle Luc::GFP expressing worms was determined at indicated times and was normalized to unstressed worms. Control worms were treated with empty vector (eV). Asterisks represent the statistical significance between the two conditions at a given time point. *P<0.05, **P<0.01, Student's *t*-test, n = 4. (C) Fluorescence micrographs of Luc::GFP in muscles at indicated times of heat stress after *hsf-1* RNAi. Control worms were treated with eV. Arrows indicate aggregates. (D) Analysis of neuronal luciferase stability in HSP1::DsRed co-expressing worms. Luciferase activity from total lysates of Luc::GFP expressing worms was determined after 3.5 h heat stress and was normalized to unstressed worms. **P<0.01, Student's *t*-test, n = 4. (E) Total luminescence of unstressed neuronal Luc::GFP expressing and HSP1::DsRed co-expressing worms. n = 3. (F) Fluorescence micrographs of Luc::GFP and HSP1::DsRed in neurons at indicated times of heat stress. Arrows assign aggregates. HSP1::DsRed accumulated in inclusions already at unstressed conditions, which did not interfere with its ability to rescue Luc::GFP from denaturation.

The tissue-specific difference in protein stability can potentially be due to differences in total chaperone levels indicating a tissue-specific divergent induction of the HSF1-mediated heat shock response. To analyze whether the higher susceptibility of neurons for stress-induced protein denaturation results from a delayed induction of the heat shock response, we investigated the activity of the *hsp-16.2* promoter under heat stress. The expression of HSP16 is strictly regulated by HSF1 and thus appears exclusively under stress conditions [12]. Employing a P*_hsp-16.2_*::GFP reporter strain, we found that within our heat stress paradigm the promoter was strongly active in muscular tissue (pharyngeal as well as body wall muscles), whereas in neurons the GFP signal appeared rather late and in a lower frequency of analyzed worms (Figure 3A). This is consistent with the analysis of other *hsp-16* promoter activities during heat stress in various tissues [12].

To link the HSF1-mediated promoter activation to our functional data, we employed RNAi experiments and analyzed *hsf-1* knock-down effects on muscular Luc::GFP. During heat stress Luc::GFP was destabilized (Figure 3B and Figure S3), resulting in a time-dependent increase of Luc::GFP aggregates (Figure 3C). Therefore, muscle cells exert an efficient HSF1-mediated heat shock response to prevent denaturation of the reporter protein. In contrast, HSF1 activity in neurons was delayed upon heat stress (Figure 3A).

The retarded neuronal heat shock response could partially be compensated by the transgenic expression of HSP1, a nematode ortholog of mammalian HSP70. Neuronal Luc::GFP was strongly stabilized and the formation of aggregates was significantly decreased during heat stress (Figures 3D, E, F). The co-expression of HSP1::DsRed did not influence total Luc::GFP levels, since the observed luminescence at control conditions was unchanged and corresponds to total levels of natively folded Luc::GFP (Figure 3E and Figure S1). This confirms that protein stability in neurons is directly coupled to total chaperone activity and emphasizes the impact of an ineffective heat shock response for protein denaturation in this tissue.

We conclude, that there is a tissue-specific hierarchy in induction of HSF1 transcriptional activity and, in particular, the neuronal heat shock response is delayed. This could be the molecular basis for the increased neuronal susceptibility to protein denaturation. In addition, the modulation of chaperone activity demonstrates that Luc::GFP stability is directly dependent on the total amount and activity of cellular chaperones.

### Analysis of tissue-specific alterations in chaperone capacity during aging of *C. elegans*

**Figure 4:**
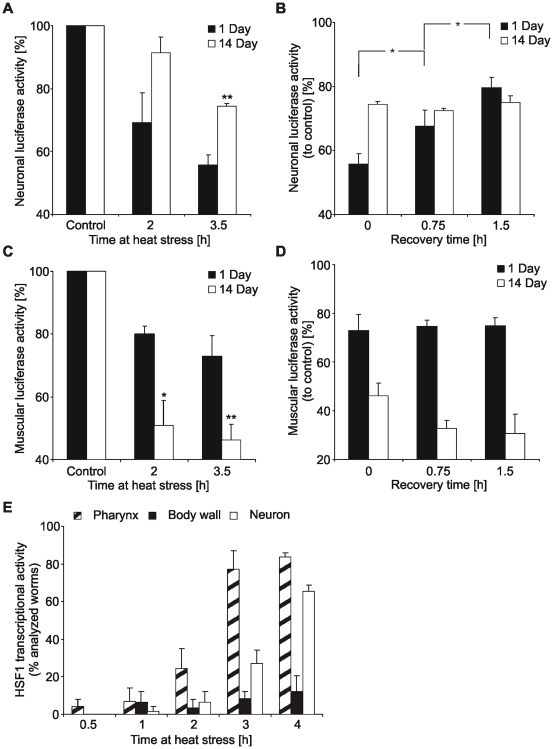
Tissue-specific alterations in chaperone capacity during aging. (A,B) Neuronal luciferase activity in young and aged worms during heat stress and subsequent recovery. Luciferase activity from total lysates of 1 and 14 day adult worms was determined at indicated times of heat stress and recovery and was compared to appropriate unstressed worms. Asterisks in (A) represent the statistical significance between young and aged worms at a given time point. *P<0.05, **P<0.01, Student's *t*-test, n = 3. (C,D) Muscular luciferase activity in young and aged worms during heat stress and subsequent recovery. Luciferase activity from total lysates of 1 and 14 day adult worms was determined at indicated times of heat stress and recovery and was compared to appropriate unstressed worms. Asterisks in (C) represent the statistical significance between young and aged worms at a given time point. *P<0.05, **P<0.01, Student's *t*-test, n = 3. (E) Analysis of HSF1 activity in aged worms, using the expression of P*_hsp-16.2_*::GFP. 10 day adult CL2070 worms were heat stressed for indicated times and the appearance of a GFP fluorescence in different tissues was analyzed in a total of at least 50 worms (n = 3). Due to an enhanced sensibility of the strain to heat stress and a reduced life span, experiments were carried out at 32°C and after 10 days of adulthood.

Instability, denaturation and aggregation of proteins are hallmarks of aging and age-associated neurodegeneration [13]. Interestingly, we observed an increased stability of neuronal Luc::GFP during heat stress in aged worms compared to young animals (Figure 4A and Figure S4). Although aged neurons were able to stabilize proteins more efficiently, they did not show a refolding activity after heat stress (Figure 4B). Aged muscle cells, in contrast, showed an increased susceptibility to protein denaturation (Figure 4C and Figure S4), which is reminiscent of a reduced chaperone activity. The absent refolding potential in muscle cells of young worms was also observed in the aged tissue (Figure 4D). These effects were independent of alterations in total Luc::GFP protein levels, since no heat-mediated changes in reporter protein levels were detected in both tissues (Figure S4). Analysis of HSF1 transcriptional activity of aged worms confirmed that the decreased protein stability in aged body wall muscle cells was associated with a deteriorated induction of the heat shock response (Figure 4E and Figure 3A). Interestingly, HSF1 activity in neurons was not strongly influenced by aging. Taken together, our data indicate that the capacity and HSF1-mediated regulation of the chaperone network is differentially affected by aging in different tissues.

## Discussion

Here we introduce a novel *C. elegans* model for investigating the activity of molecular chaperones *in vivo* and provide insights into characteristic tissue- and age-specific differences in chaperoning. The reporter protein Luc::GFP was chosen to evaluate the degree of protein denaturation and the subsequent refolding by analyzing the enzymatic activity of luciferase, by imaging of GFP, and by immunoblotting of aggregates. Importantly, we found a clear correlation between the stability of Luc::GFP and the activity of cellular chaperones, which provides direct information about the total chaperone capacity in the particular tissue analyzed.

Different cell types have distinct requirements for their particular chaperone network. Even if the major components are almost identical in different tissues, the levels of certain chaperones or isoforms vary, which may influence the total capacity of the accordant chaperone system [14]. The exact strategies of cellular chaperoning are not well-known to date. Here, we depict two tissue-specific systems, which show differential effects during heat stress. Muscle cells efficiently stabilize proteins, whereas neuronal cells are highly prone for protein denaturation. However, the neuronal defence system rather focuses on the potent and rapid renaturation or disaggregation of misfolded proteins.

Importantly, the enhanced stability of Luc::GFP in muscle cells was dependent on the activity of HSF1, as was shown by *hsf-1* knock down, demonstrating that a functional heat shock response is indeed essential for the observed protein stability. The induction of HSF1 transcriptional activity followed a tissue-specific hierarchy and, in particular, the neuronal heat shock response was decelerated. Interestingly, also for differentiated neurons of higher eukaryotes it was shown that the induction of HSPs following heat stress is delayed or absent and results from a high activation threshold of the heat shock response [15]–[18]. During stress conditions monomeric, inactive HSF1 translocates into the nucleus, trimerizes, binds to heat shock response elements and becomes phosphorylated at multiple sites. This phosphorylation leads to the recruitment of the transcriptional machinery to the promoter and finally results in transactivation of *hsp* genes [19], [20]. Thus, the delayed induction of the heat shock response in neurons of *C. elegans* could occur at the HSF1 expression level, its trimerization efficiency and promoter binding or activation by phosphorylation and could be the molecular basis for the increased neuronal susceptibility to protein denaturation.

In aged muscle cells protein stability is basically reduced, which is associated with a strongly decreased heat shock response. In contrast, aged neurons are more resistant to heat stress than their young counterparts, but they lost the characteristic refolding potential. Thus, aged neurons display a chaperone activity that is identical to young muscle cells, but unlike young muscle cells the increased protein stability is independent of a potent induction of the heat shock response. This interesting age-related “*change of chaperoning strategy*” is potentially resulting from the susceptibility of neuronal cells to protein aggregation and could be of importance for the aggregate clearance efficacy during neurodegeneration due to protein conformational diseases.

In conclusion, the analysis of tissue- and age-specific characteristics of chaperoning revealed significant alterations comparing neurons and muscle cells of *C. elegans* and might be important for the development of novel strategies for prevention or delay of aberrant protein folding that may lead to neurodegenerative disorders. In particular, difficulties to activate the neuronal heat shock response and alterations in the neuronal protein folding efficiency during aging are of substantial interest, since for many neurodegenerative diseases age is the major risk factor and the exact age-associated changes increasing disease frequency are not well-established to date.

## Materials and Methods

### Nematode strains

*C. elegans* were maintained at 20°C using standard procedures [21]. To generate transgenic lines, expression vectors together with the co-injection marker pRF4 [rol-6 (su1006)] were injected into WT N2 (Bristol) worms at 60 µg/ml each. For integration, worms were UV treated and integrated lines were outcrossed for at least 4 times. For each transgene 2 independent lines were generated and analyzed. The strain CL2070, dvIs70 [P*_hsp16.2_*::gfp; rol6(su1006)] [22] was obtained from the *Caenorhabditis* Genetic Center.

### Plasmid constructs

Plasmids were constructed using the Gateway Technology (Invitrogen). To generate promoter::luc::gfp constructs, the accordant promoter was PCR amplified from genomic DNA and luciferase cDNA was amplified from the plasmid pGL3 (Promega). Both amplificates were fused using the fusion PCR protocol of Hobert, 2002 [23]. The fusions were shuttled from an entry vector (pCR8, TOPO-TA Cloning Kit, Invitrogen) into the destination vector pRL1899 (kind gift of Rueyling Lin, UTSW, Dallas) containing a 3′ GFP and unc-54 UTR. The molecular cloning yielded expression plasmids: P*_sng-1_*::luc::gfp, P*_myo-3_*::luc::gfp and P*_ehs-1_*::hsp-1::DsRed-N2. The latter construct was generated virtually identically, except for DsRed being amplified from the vector pDsRed-N2 (Clontech) and that the fusion PCR was carried out with all 3 primary amplificates together in one reaction.

### Evaluation of tissue area

For evaluation of total body wall muscle and neuronal tissue area 5 nematodes of each line were imaged using a confocal fluorescence microscope (LSM 710, Zeiss). For each image the same microscope settings were employed to simplify comparison and to avoid overexposure. Worms were analyzed at 1 µm thick Z-sections for a total of 35–40 slices. Subsequently, the area of GFP fluorescence throughout the stack was evaluated using the ImageJ software for area determination.

### Heat Stress and luminescence measurement

Luc::GFP expressing worms were heat stressed at 35°C (±1°C) on NGM plates in an incubator. At the indicated times, a duplicate or triplicate of 3–6 worms each was immediately shock frozen in liquid nitrogen in 100 µl reporter lysis buffer (Promega). After thawing, the samples were shortly sonicated (50 Hz), incubated on ice for 10 min and transferred into single wells of a black 96-well dish. 50 µl of ATP buffer (100 mM KH_2_PO_4_, 5 mM ATP, 10 mM MgCl_2_) were added and the luminescence was measured using the Victor Multilabel Plate Reader (PerkinElmer) after dispensing 100 µl of 0.02 mM luciferin (Synchem). For recovery experiments worms were heat stressed at 35°C for 3.5 h and afterwards incubated at 20°C for indicated times. Since CL2070 worms were more vulnerable to heat stress and showed a reduced life span compared to Luc::GFP and N2 worms we adapted the experimental conditions for this strain to 32°C heat stress and 10 days of adulthood for aged worms. At these conditions CL2070 worms displayed morphological and phenotypical characteristics and mortality rates identical to Luc::GFP strains.

### Fluorescence microscopy

Worms were mounted on 2% agar pads on a glass slide, immobilized in 100 mg/ml levamisole and fluorescence was viewed on an Olympus IX81. Images were captured with a FVII camera using CelˆR imaging software.

### Immunoblotting

5 to 10 worms were transferred into 2× gel loading buffer, sonified with 3 short strokes (50 Hz) and incubated for 5 min at 95°C. The whole sample was loaded onto precast NuPAGE Bis-Tris gels (Invitrogen) and detected using following antibodies: α-luciferase (Zymed), α-tubulin (Sigma), α -HSP70 #975 (kind gift of Dr F.U. Hartl).

### Preparation of protein aggregates

Aggregates were prepared as previously described [24]. Shortly, 60–80 worms were extracted in reporter lysis buffer containing protease inhibitor cocktail (Sigma) and sonicated 4× at 50 Hz for 5 sec. Protein aggregates were pelleted at 15.500 *g* for 15 min. The supernatant was collected (S) and the pellet was washed, resuspended in SDS-containing lysis buffer and sonicated 4× at 50 Hz (P). All samples were incubated for 5 min at 95°C and same protein amounts were loaded onto NuPAGE Bis-Tris gels.

### RNA interference

RNAi was induced by feeding nematodes double stranded RNA of the target gene *hsf-1* using the RNAi clone obtained from the Ahringer RNAi library [25]. The experiments were conducted using protocols according to standard procedures. RNAi plates consisted of NGM supplemented with 1 mM β-D-isothiogalactopyranoside and 100 µg/ml ampicillin. L4 worms were transferred on RNAi plates seeded with the RNAi clone or the L4440 (Addgene) empty vector control and were daily removed to fresh plates. After 72 h worms were employed for heat stress experiments as described above.

### Statistical methods

Statistical significance was determined by Student's *t*-test using SIGMA STAT software (SPSS Science). The results are depicted as mean ± standard deviation.

## Supporting Information

Figure S1 Luc::GFP protein levels and total luciferase activity. (A) Immunoblotting of total Luc::GFP protein levels in neuronal and muscle tissue. For detection of Luc::GFP an antibody directed against luciferase was used. Tubulin served for loading control. (B) Evaluation of neuronal and muscular luciferase activity from total lysates of an increasing number of Luc::GFP expressing worms (n = 3). Total protein levels of Luc::GFP were approximately 9 times higher in muscle cells compared to neuronal cells. Determination of luciferase activities resulted in an approximately 10 times increased activity for muscle cells. Due to tissue-specific differences in reporter protein levels and total tissue volume, we evaluated the corresponding ratio of Luc::GFP levels to total tissue area to exclude reporter protein concentration effects on protein denaturation or aggregation. The calculated area of muscle cell tissue was approximately 3.4 times higher than the area of neuronal tissue. This resulted in an approximately 2.6 times increased expression density of Luc::GFP in muscle cells compared to neuronal cells. (C) Analysis of Luc::GFP protein levels during heat stress. Luc::GFP expressing worms were heat stressed at 35°C for 3.5 h and protein levels were analyzed by immunoblotting. For detection of Luc::GFP an antibody directed against luciferase was used. Tubulin served for loading control. Graphical representations of the ratio Luc::GFP to tubulin were calculated using optical band densities. n = 3.
(9.40 MB TIF)

Figure S2 Inhibition of protein translation using cycloheximide. The successful inhibition of protein translation by cycloheximide was demonstrated by analyzing the induction of HSP70 isoforms during heat stress. Worms were pre-incubated with 0.6 mg/ml cycloheximide (CHX) or M9 buffer (V) for 15 min and heat stressed at 35°C. At indicated times 5 worms were transferred into 2x gel loading buffer and immediately shock frozen. The whole sample was loaded onto NuPAGE Bis-Tris gels and HSP70 isoforms were detected by an antibody directed against HSP70. Tubulin served for loading control. Graphical representations of the ratio HSP70 to tubulin were calculated using optical band densities. *P<0.05, Student's t-test, n = 4.
(4.01 MB TIF)

Figure S3 hsf-1 RNAi successfully decreases hsf-1 mRNA levels and does not affect luciferase activity. (A) Total levels of hsf-1 mRNA were evaluated using real time PCR. Worms were treated with hsf-1 RNAi or empty vector (eV) for 72 h and RNA was extracted using the Absolutely RNA Miniprep Kit (Stratagene). Reverse transcription was performed on 0.5 µg total RNA using the Omniscript RT Kit (Qiagen) and 1 µM oligo(dT)23primer (Sigma) according to the manufacturer's instructions. Real-time PCR was carried out in a 25 µl reaction volume containing 1 µl cDNA, 0.5 µl sense and antisense primer (100 pmol) and 12.5 µl of 2x Absolute SYBR Green Fluorescein Mix (Abgene) using the iCycler Real-Time Thermocycler (Biorad). The following oligos were used: hsf-1 forward 5′-GAAATGTTTTGCCGCATTTT-3′, hsf-1 reverse 5′-CCTTGGGACAGTGGAGTCAT-3′; rpl-21 (reference gene) forward 5′-CCAGTCCCAGCTTTGAAGAG-3′, rpl-21 reverse 5′-ACAATCTCGAAACGGAGTGG-3′. After an initial 15 min denaturation/activation step, 35 PCR cycles were carried out. PCR conditions were 95°C for 20 sec, 60°C for 20 sec and 72°C for 30 sec. The PCR cycle number that generated the first fluorescence signal above threshold was determined. Specificity of the reaction was confirmed by melting curve analysis. (B) Luc::GFP levels in muscle cells are not influenced by hsf-1 RNAi. To analyze whether the activity of Luc::GFP from muscle cells is altered by the hsf-1 knock-down, we compared the luminescence from total worm lysates of unstressed worms after 72 h of eV and RNAi treatment (n = 3). The luminescence corresponds to total protein levels of natively folded Luc::GFP (Fig. S1).
(2.68 MB TIF)

Figure S4 Aging decreases total luciferase activity and heat stress does not affect Luc::GFP protein levels. (A) Analysis of total luciferase activity from lysates of unstressed young and aged Luc::GFP expressing worms. The total luminescence was reduced in both aged tissues compared to the appropriate young tissues (neuron: ∼33%, muscle: ∼38%; n = 3). The luminescence corresponds to total protein levels of natively folded Luc::GFP (Fig. S1). (B) Analysis of Luc::GFP protein levels during heat stress. 14 day adult Luc::GFP expressing worms were heat stressed at 35°C for 3.5 h and Luc::GFP protein levels were analyzed by immunoblotting. For detection of Luc::GFP an antibody directed against luciferase was used. Tubulin served for loading control. Graphical representations of the ratio Luc::GFP to tubulin were calculated using optical band densities. n = 3.
(8.14 MB TIF)
