# Supplementary figures and images for: HSF1-Controlled and Age-Associated Chaperone Capacity in Neurons and Muscle Cells of C. elegans
Source: PLoS One. 2010 Jan 5;5(1):e8568. doi: 10.1371/journal.pone.0008568 (PMC2797298; doi:10.1371/journal.pone.0008568)

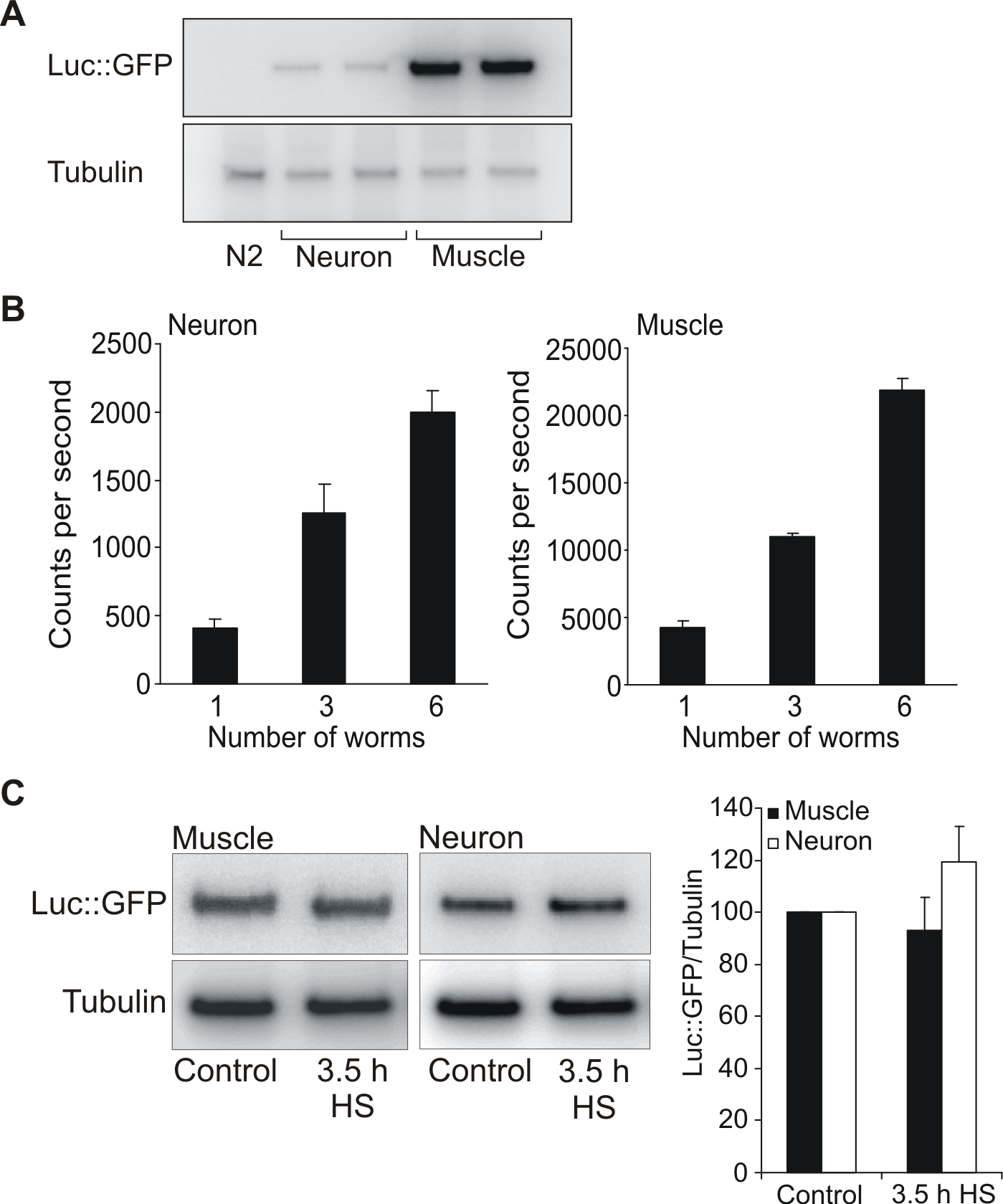

Supplement: Figure S1 — Luc::GFP protein levels and total luciferase activity. (A) Immunoblotting of total Luc::GFP protein levels in neuronal and muscle tissue. For detection of Luc::GFP an antibody directed against luciferase was used. Tubulin served for loading control. (B) Evaluation of neuronal and muscular luciferase activity from total lysates of an increasing number of Luc::GFP expressing worms (n = 3). Total protein levels of Luc::GFP were approximately 9 times higher in muscle cells compared to neuronal cells. Determination of luciferase activities resulted in an approximately 10 times increased activity for muscle cells. Due to tissue-specific differences in reporter protein levels and total tissue volume, we evaluated the corresponding ratio of Luc::GFP levels to total tissue area to exclude reporter protein concentration effects on protein denaturation or aggregation. The calculated area of muscle cell tissue was approximately 3.4 times higher than the area of neuronal tissue. This resulted in an approximately 2.6 times increased expression density of Luc::GFP in muscle cells compared to neuronal cells. (C) Analysis of Luc::GFP protein levels during heat stress. Luc::GFP expressing worms were heat stressed at 35°C for 3.5 h and protein levels were analyzed by immunoblotting. For detection of Luc::GFP an antibody directed against luciferase was used. Tubulin served for loading control. Graphical representations of the ratio Luc::GFP to tubulin were calculated using optical band densities. n = 3. (9.40 MB TIF) [file pone.0008568.s001.tif]

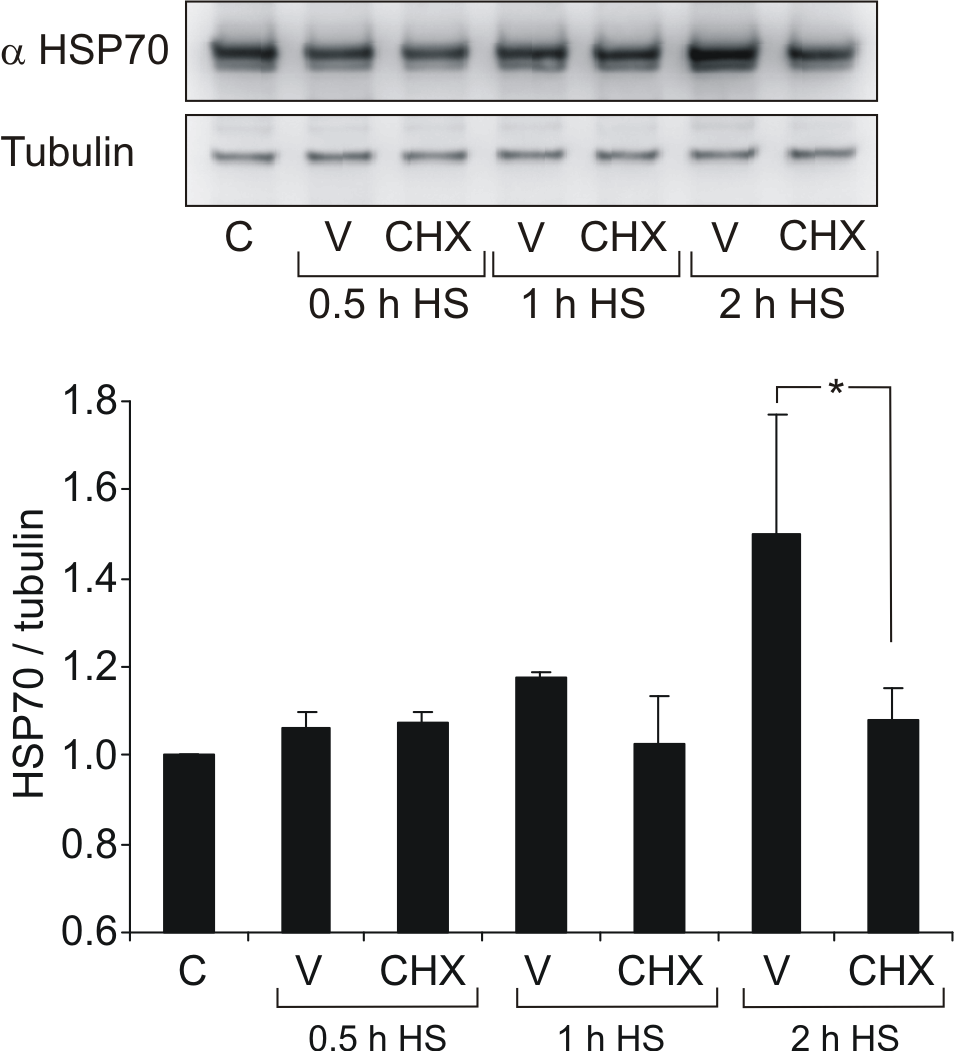

Supplement: Figure S2 — Inhibition of protein translation using cycloheximide. The successful inhibition of protein translation by cycloheximide was demonstrated by analyzing the induction of HSP70 isoforms during heat stress. Worms were pre-incubated with 0.6 mg/ml cycloheximide (CHX) or M9 buffer (V) for 15 min and heat stressed at 35°C. At indicated times 5 worms were transferred into 2x gel loading buffer and immediately shock frozen. The whole sample was loaded onto NuPAGE Bis-Tris gels and HSP70 isoforms were detected by an antibody directed against HSP70. Tubulin served for loading control. Graphical representations of the ratio HSP70 to tubulin were calculated using optical band densities. *P<0.05, Student's t-test, n = 4. (4.01 MB TIF) [file pone.0008568.s002.tif]

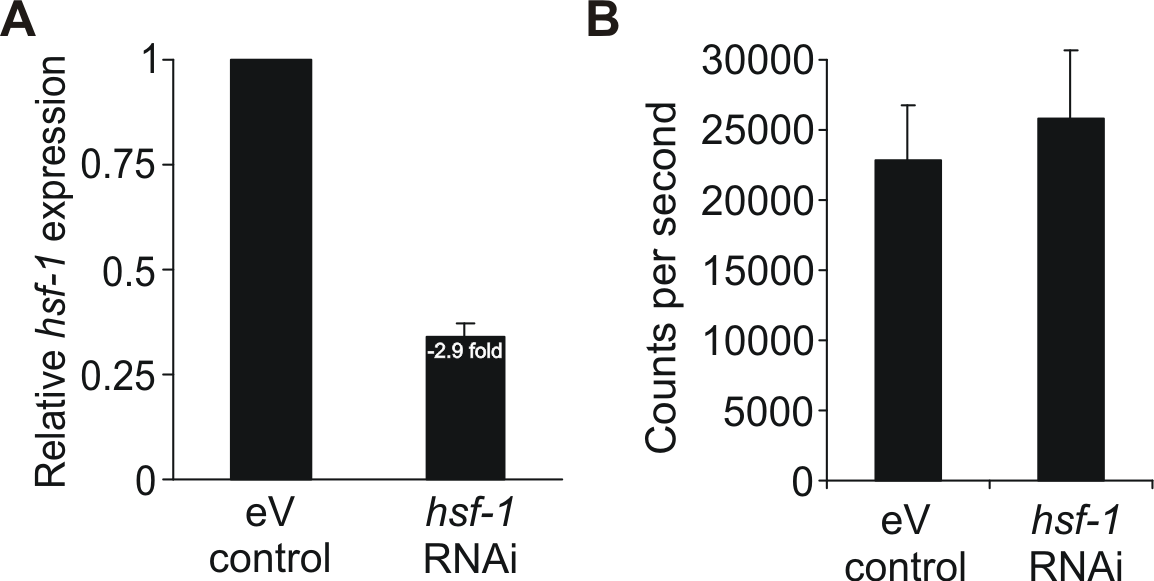

Supplement: Figure S3 — hsf-1 RNAi successfully decreases hsf-1 mRNA levels and does not affect luciferase activity. (A) Total levels of hsf-1 mRNA were evaluated using real time PCR. Worms were treated with hsf-1 RNAi or empty vector (eV) for 72 h and RNA was extracted using the Absolutely RNA Miniprep Kit (Stratagene). Reverse transcription was performed on 0.5 µg total RNA using the Omniscript RT Kit (Qiagen) and 1 µM oligo(dT)23primer (Sigma) according to the manufacturer's instructions. Real-time PCR was carried out in a 25 µl reaction volume containing 1 µl cDNA, 0.5 µl sense and antisense primer (100 pmol) and 12.5 µl of 2x Absolute SYBR Green Fluorescein Mix (Abgene) using the iCycler Real-Time Thermocycler (Biorad). The following oligos were used: hsf-1 forward 5′-GAAATGTTTTGCCGCATTTT-3′, hsf-1 reverse 5′-CCTTGGGACAGTGGAGTCAT-3′; rpl-21 (reference gene) forward 5′-CCAGTCCCAGCTTTGAAGAG-3′, rpl-21 reverse 5′-ACAATCTCGAAACGGAGTGG-3′. After an initial 15 min denaturation/activation step, 35 PCR cycles were carried out. PCR conditions were 95°C for 20 sec, 60°C for 20 sec and 72°C for 30 sec. The PCR cycle number that generated the first fluorescence signal above threshold was determined. Specificity of the reaction was confirmed by melting curve analysis. (B) Luc::GFP levels in muscle cells are not influenced by hsf-1 RNAi. To analyze whether the activity of Luc::GFP from muscle cells is altered by the hsf-1 knock-down, we compared the luminescence from total worm lysates of unstressed worms after 72 h of eV and RNAi treatment (n = 3). The luminescence corresponds to total protein levels of natively folded Luc::GFP (Fig. S1). (2.68 MB TIF) [file pone.0008568.s003.tif]

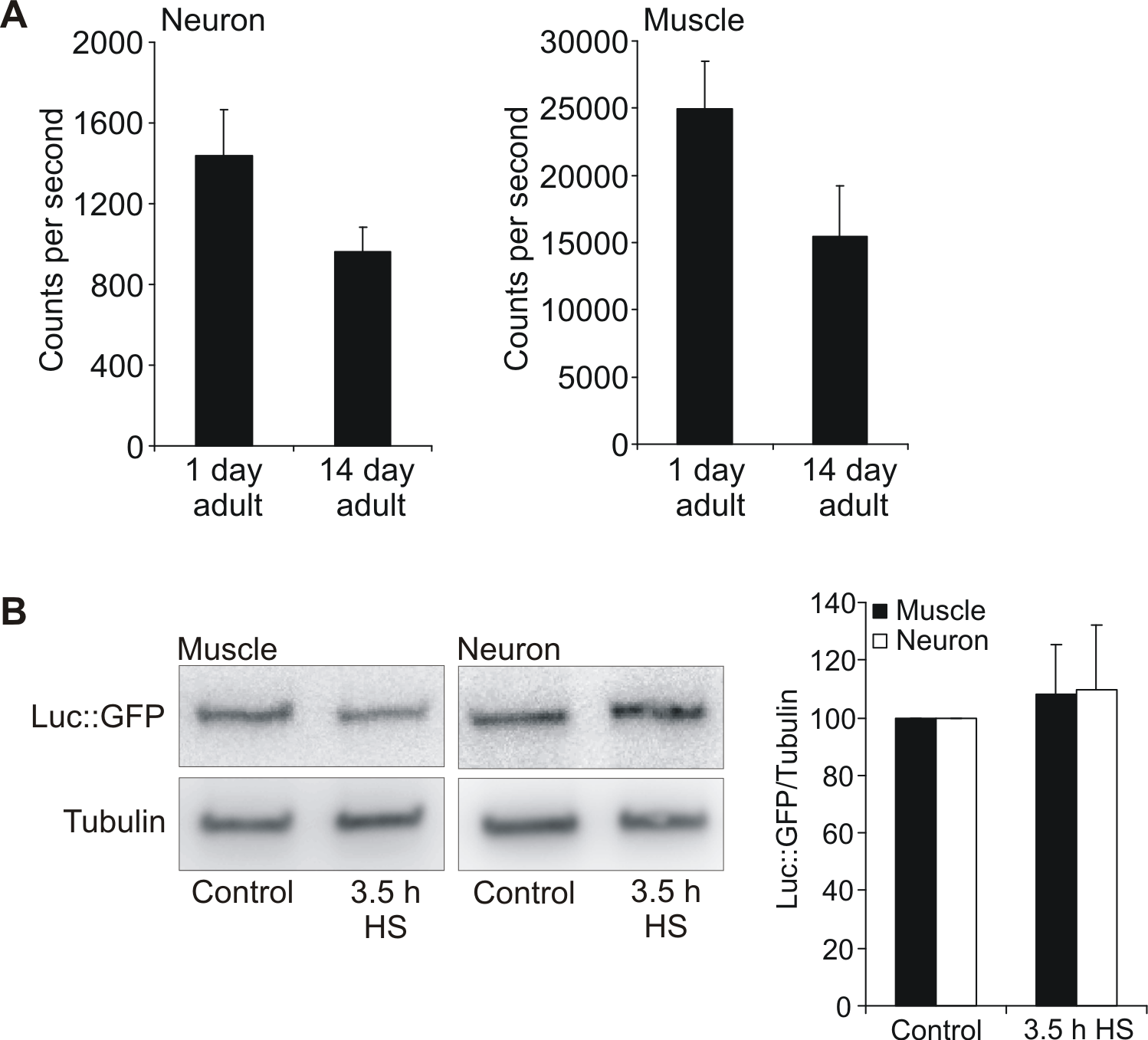

Supplement: Figure S4 — Aging decreases total luciferase activity and heat stress does not affect Luc::GFP protein levels. (A) Analysis of total luciferase activity from lysates of unstressed young and aged Luc::GFP expressing worms. The total luminescence was reduced in both aged tissues compared to the appropriate young tissues (neuron: ∼33%, muscle: ∼38%; n = 3). The luminescence corresponds to total protein levels of natively folded Luc::GFP (Fig. S1). (B) Analysis of Luc::GFP protein levels during heat stress. 14 day adult Luc::GFP expressing worms were heat stressed at 35°C for 3.5 h and Luc::GFP protein levels were analyzed by immunoblotting. For detection of Luc::GFP an antibody directed against luciferase was used. Tubulin served for loading control. Graphical representations of the ratio Luc::GFP to tubulin were calculated using optical band densities. n = 3. (8.14 MB TIF) [file pone.0008568.s004.tif]
